# Natural based hydrogels promote chondrogenic differentiation of human mesenchymal stem cells

**DOI:** 10.3389/fbioe.2024.1363241

**Published:** 2024-03-19

**Authors:** Tina Zahedi Tehrani, Shiva Irani, Abdolreza Ardeshirylajimi, Ehsan Seyedjafari

**Affiliations:** ^1^ Department of Biology, Science and Research Branch, Islamic Azad University, Tehran, Iran; ^2^ Urogenital Stem Cell Research Center, Shahid Beheshti University of Medical Sciences, Tehran, Iran; ^3^ Department of Biotechnology, College of Science, University of Tehran, Tehran, Iran

**Keywords:** Chitosan, alginate, hydrogel, cartilage tissue engineering, mesenchymal stem cell

## Abstract

**Background:** The cartilage tissue lacks blood vessels, which is composed of chondrocytes and ECM. Due to this vessel-less structure, it is difficult to repair cartilage tissue damages. One of the new methods to repair cartilage damage is to use tissue engineering. In the present study, it was attempted to simulate a three-dimensional environment similar to the natural ECM of cartilage tissue by using hydrogels made of natural materials, including Chitosan and different ratios of Alginate.

**Material and methods:** Chitosan, alginate and Chitosan/Alginate hydrogels were fabricated. Fourier Transform Infrared, XRD, swelling ratio, porosity measurement and degradation tests were applied to scaffolds characterization. After that, human adipose derived-mesenchymal stem cells (hADMSCs) were cultured on the hydrogels and then their viability and chondrogenic differentiation capacity were studied. Safranin O and Alcian blue staining, immunofluorescence staining and real time RT-PCR were used as analytical methods for chondrogenic differentiation potential evaluation of hADMSCs when cultured on the hydrogels.

**Results:** The highest degradation rate was detected in Chitosan/Alginate (1:0.5) group The scaffold biocompatibility results revealed that the viability of the cells cultured on the hydrogels groups was not significantly different with the cells cultured in the control group. Safranin O staining, Alcian blue staining, immunofluorescence staining and real time PCR results revealed that the chondrogenic differentiation potential of the hADMSCs when grown on the Chitosan/Alginate hydrogel (1:0.5) was significantly higher than those cell grown on the other groups.

**Conclusion:** Taken together, these results suggest that Chitosan/Alginate hydrogel (1:0.5) could be a promising candidate for cartilage tissue engineering applications.

## 1 Introduction

Hyaline cartilage, also called articular cartilage is a connective tissue in nature which mainly consists of chondrocytes and extracellular matrix (ECM) containing abundant collagen fibers and proteoglycans (Ortiz-Arrabal et al., 2021; Wu et al., 2021). The self-healing capability of damaged cartilage tissue is notably bounded due to features such as avascularity, neural and lymphatic characteristics, the poor migration ability of chondrocytes, and the small number of progenitor cells (Ortiz-Arrabal et al., 2021; Guo et al., 2023), which may cause structural anomalies and malfunction(Redondo et al., 2018). Common surgical methods contain abrasion arthroplasty, drilling, and micro-fracture, but these techniques do not yield ideal results(Mobasheri et al., 2014), and the repair of cartilage defects is still a challenge for clinical surgeons (Kwon et al., 2019; Xue et al., 2021).

It seems that cartilage tissue engineering is a promising and efficient cure for classic treatment strategies. Its goals are to recreate cartilaginous substitutes with the same features as natural cartilage (Messaoudi et al., 2020; Rezaei et al., 2020).

The most natural source of cells for stimulating the repair of damaged cartilage is chondrocytes. However, its clinical application in cartilage tissue engineering is limited due to the chondrocytes' little proliferation and dedifferentiation after long *in vitro* development (Huch et al., 2002). Unlike chondrocytes, mesenchymal stem cells (MSCs) can be isolated from various tissues, such as bone, bone marrow, fat, and synovial membrane and fluid (Rezaei et al., 2020). MSCs have been thoroughly investigated as cell origins for cartilage tissue engineering because of their ability to adhere to and mature on plastic and differentiate into chondrocytes and other lineages such as adipocytes and osteoblasts (tri-lineage potential) under conditions optimized for single lineages (Nakayama et al., 2021; Thorp et al., 2021). The base of human tissue-like substitute manufacturing in tissue engineering is the mixture of human cells with biocompatible biomaterials as scaffolds (Ortiz-Arrabal et al., 2021). Ideally, biomaterials used in tissue engineering should mimic the structure and biological features of the natural ECM, aid cell adhesion, and raise tissue modification (Ortiz-Arrabal et al., 2021). One kind of scaffold used in cartilage tissue engineering is hydrogel. A combination of MSCs and hydrogels can reach the defect area of the cartilage and promote cartilage regeneration. Hydrogels possess cartilage tissue-like characteristics (Wei et al., 2021); have flexibility and adaptation in fabrication, diversity in composition, high plasticity in shape, excellent biocompatibility (20), and suitable pore size and porosity (Bao et al., 2020); and their 3D hydrophilic polymer networks are similar to the articular cartilage extracellular matrix (Mokhames et al., 2020; Li et al., 2021). Natural materials used as hydrogels are biocompatible and biodegradable and their degradation does not generate toxic and immunogenic products (Bao et al., 2020). They also have a highly hydrated viscoelastic matrix and tunable swelling behavior. Alginate (Alg) and chitosan (Chi) are potential natural polysaccharides for cartilage repair (Duan et al., 2023). Alg, isolated from the cell wall of brown algae possesses biodegradability, high biocompatibility, availability, and cell-friendly gelation. These features explain its wide application (Zhao et al., 2021). Chi is another natural polymer isolated from chitin and used in tissue engineering (TE) because of its biocompatibility, *in vivo* degradation and antimicrobial features, non-antigenicity, adsorption abilities, and (Mokhames et al., 2020) lack of complications, such as inflammation or allergic reactions after implantation (Zhao et al., 2021).

In the present study hypothesized that the performance of Chi/Alg hydrogels in cartilage regeneration could be different with various ratios of alginate in the Chi hydrogel. The fabricated Chi/Alg hydrogels were characterized by morphological analyses, FTIR (Fourier Transform Infrared), and XRD (X-ray powder diffraction). The compressive strength test, swelling rate, and degradation rate were performed to confirm the mechanical properties of Chi/Alg hydrogels. Furthermore, in addition to the toxicity study *via* MTT assay chondrogenic differentiation potential of the human adipose derived MSCs (hADMSCs) was evaluated using alcian blue and safranin-O staining, immunofluorescence staining, and real-time RT-PCR.

## 2 Materials and methods

### 2.1 Materials

Sodium alginate pharmaceutical grade and high purity (PN-180947), chitosan Pharmaceutical grade high purity medium molecular weight (PN-448877), and calcium chloride(CaCl_2_) (PN-C1016) were all purchased from Sigma-Aldrich (St. Louis)., dimethylsulfoxide (DMSO), 4′,6-diamidino-2-phenylindole (DAPI), 3-(4,5-dimethylthiazol-2-yl)-2,5-diphenyl tetrazolium bromide (MTT) were purchased from Sigma-Aldrich, St. Louis MO. Trypsin/EDTA solution (0.25%), phosphate-buffered saline (PBS), fetal bovine serum (FBS), High glucose Dulbecco’s minimum essential medium (DMEM), antibiotics (penicillin/streptomycin), 100 units/mL were obtained from Gibco, Burlington, ON, Canada. Acetic acid, sodium hydroxide (NaOH), ethanol, isopropanol, chloroform, dimethylformamide (DMF), glutaraldehyde, ARS, and Triton 100X were all bought from Merck, Darmstadt, Germany. Lipase *pseudomonas cepacia* (Grade 62,309-100 mg) was procured from Sigma, Aldrich, St. Louis.

### 2.2 Methods

#### 2.2.1 Scaffolds fabrication

##### 2.2.1.1 Alginate scaffold

For alginate hydrogel fabrication (1%w/v), 1 g sodium alginate was solved in 100 mL of double distilled water. The sodium alginate solution was stirred for 30 min via magnetic stirring with 100 mM of CaCl_2_ to make characteristic hydrogels. The finalized alginate hydrogel was prepared by centrifugation and removal of the supernatant.

##### 2.2.1.2 Chitosan scaffolds

For chitosan hydrogel fabrication (1% w/v), 1 g of chitosan powder was solved in 100 mL of acetic acid (2% v/v). Then 0.3 mL glutaraldehyde solution was combined with chitosan solution and mixed *via* magnetic stirring to obtain homogeneous solution.

##### 2.2.1.3 Chitosan/alginate scaffolds

Firstly, for making a homogeneous solution, Alginate (0.25%, 0.5%, 1% w/v) was solved in 100 mL of water *via* employing a mechanical stirrer (RW 20.n Lobortechik, Wasserburg, Germany) for 1 h. Secondly, dissolved chitosan (1% w/v, 310 kDa, and 90%) in 50 mL of 2% acetic acid solution was carefully combined with the alginate solution using a dropper. The homogeneous gel solution was stirred at 500 rpm for 1–2 h at room temperature. This gel solution was transmitted into the tissue culture plate (35 × 10 mm^2^), frozen at −24°C for 1 day, and freeze-dried to form scaffolds. These scaffolds were immersed or cross-linked with 10% CaCl_2_ solution for 30 min, then soaked in absolute ethyl alcohol for 10 min. Finally, scaffolds were washed with a large amount of water and freeze-dried again. In the present study, we have used Chi and Alg in a 3 different weight ratios (1:0.25, 1:0.5, and 1:1) for the Chi-Alg scaffold construction.

#### 2.2.2 Scaffolds characterization

##### 2.2.2.1 Scanning electron microscopy

The morphology of the hydrogels and the pore sizes were studied using a scanning electron microscope (SEM, XL30 model, Philips). Briefly, dried scaffold samples were cut into little segments sputter-coated with gold to a width of 200–500 Å and placed on a metal stub for observation under SEM. Examination of the SEM images for estimating the pore size and porosity percentage of the scaffolds was carried out using ImageJ^®^ software. The values are presented as mean ± standard deviation.

##### 2.2.2.2 Attenuated total Reflectance-Fourier Transform Infrared (ATR-FTIR) spectroscopy and X-ray powder diffraction (XRD)

The chemical combination of the scaffolds was determined using ATR-FTIR spectroscopy and XRD (ARL X’TRA, Thermo Electron, United States). Fracture data were collected in a range of 2θ from the accepted manuscript up to 80° using monochromatic CuKα radiation. Digital images of the made scaffolds were recorded with a camera (Nikon D3100, Nikon Corporation).

##### 2.2.2.3 Compression test

The compressive moduli of the manufactured scaffolds (6 mm in diameter and 12 mm in height) were determined using a strength measuring device made by Santam KN 25 according to ASTM F451-99a standard 100N once at a speed of 1 mm/min, up to a maximum pressure of 80%.

##### 2.2.2.4 Swelling ratio

In this test, to determine the water absorption capacity of the hydrogels, their initial weights were calculated after freeze-drying (Wd). The hydrogels were then immersed in PBS at room temperature for 1, 2, 4, 6, and 24 h. In the next step, the excess water of hydrogels was absorbed with a paper towel. Subsequently, they were placed inside the oven for equal removal of excess water from all samples at 40°C for 10 s, and their wet weights (Ww) were measured. Then SR was determined using the following formula. The values are reported as mean ± standard deviation (n = 3).

$$Swelling\;ratio\left(\%\right)=\left[\frac{W_{w}-W_{d}}{W_{d}}\right]\times 100$$

where W_d_ and W_w_ are the main weight and the wet weight of specimens, respectively.

##### 2.2.2.5 Degradation test

The *in-vitro* degradation rate of the samples was studied using the degradation test. The initial weight of each freeze-dried hydrogel was measured before soaking in PBS and PBS-containing lipase (w). Then samples were soaked in PBS (0.01 M, pH = 7.4) and PBS-lipase at 37 °C with a rotational speed of 28 rpm (Thermoshaker, LS-100, Thermo Scientific, United States) for 21 days. PBS and PBS-lipase were replaced every 3 days. The lipase enzyme prepared from *Pseudomonas cepacia* was solved in PBS at a concentration of 0.5 mg/mL. The scaffolds were washed with deionized water to remove the remaining salts, dried with filter paper, and then oven-dried for 24 h. The dry weights of the scaffolds were noted as (Wt). The degradation weight of each scaffold was calculated as stated in the equation below:

$$Weight\;loss\left(\%\right)=\left[\frac{W-W_{t}}{W}\right]\times 100$$

##### 2.2.2.6 Porosity measurement

The total porosity was defined *via* the liquid displacement method. At first, the dry weight of the hydrogels and the mass of ethanol were evaluated. Next, the hydrogels were soaked in pure alcohol for 48 h until the alcohol was absorbed and saturated in them, and then they were re-weighed. Finally, the porosity of the hydrogels was estimated using the following formula:

where V_1_ is the volume of the scaffold immersed in a graduated container filled with ethanol, V_2_ is the volume of total ethanol and the submerged scaffolds, and V_3_ is the volume of residual ethanol after the removal of the scaffold.

The total volume of the hydrogels was measured using the following equation:

$$v=\left(v_{2}-v_{1}\right)+\left(v_{1}-v_{3}\right)=v_{2}-v_{3}$$

In this equation, the primary volume of hydrogels is (v_2_-v_1_), and the volume of ethanol absorbed by the hydrogels is (v_1_-v_3_). Finally, the hydrogel’s porosity rate was evaluated using the following equation:

$$Porosity=\frac{\left(v_{1}-v_{3}\right)}{\left(v_{2}-v_{3}\right)}$$

#### 2.2.3 *In-vitro* cell culture, proliferation and biocompatibility

##### 2.2.3.1 Scaffold sterilization and cell culture

In this study, freeze-dried hydrogels were divided into small segments and soaked in 70% ethanol for 1 h in 24-well plates. Afterward, they were washed with sterilized PBS containing 1% antibiotics (penicillin-streptomycin) three times for 5 min to clear the residual ethanol from the samples. Finally, each side of the hydrogels was sterilized with ultraviolet radiation for 30 min. Then, the sterilized scaffolds were immersed in Dulbecco’s modification of Eagle medium (DMEM, Gibco) for cell culture preparation and incubated at 37 °C, 5% CO_2_, and 95% air. The cell culture medium was removed after a 24-h incubation, and hADMSCs at the third passage purchased from the Iranian Biological Resource Center were seeded onto the hydrogels at a density of 1 × 10^4^ cells/well. A polystyrene tissue culture plate (TCP), which was seeded with the same density of cells, was used as the control group. Afterward, the complete cell culture medium, including 89% DMEM, 10% fetal bovine serum (FBS, Gibco), and 1% penicillin/streptomycin (Gibco), was added to the wells. The plates were placed in a cell culture incubator. The culture medium was also replaced every 3 days.

##### 2.2.3.2 Scaffold biocompatibility

The 3-(4,5-dimethylthiazol-2-yl)-2,5-diphenyl tetrazolium bromide (MTT) assay was carried out on the 1st, 3rd, and 7th days of the test. The samples were rinsed with PBS, and the culture medium was changed with 200 μL DMEM containing 5 mg/mL MTT, and then incubated for 4 h at 37°C. After incubating, dimethyl sulfoxide (DMSO, 100 μL) was replaced with a culture medium to dissolve the purple formazan crystals in living cells. For better MTT residue solvation, the plate was located on a shaker for 15 min. Finally, cell proliferation and optical densities were measured at 570 nm using a microplate reader (ELx 800, BioTek). The values are reported as mean ± standard deviation (n = 3).

#### 2.2.4 Chondrogenic differentiation

The hAMSCs seeded scaffolds were cultured under a chondrogenic medium containing high glucose (4.5 g/L) DMEM supplemented with 10% FBS, 1% PS, 1% insulin-transferrin selenium (ITS, Thermo Fisher Scientific, Waltham, MA, United States), 350 μML-proline (Carl Roth GmbH, Karlsruhe, Germany), 100 nM dexamethasone, 170 µM ascorbic acid-phosphate (Sigma Aldrich, St. Louis, MO, United States), and 10 ng/mL TGF-β3 (Thermo Fisher Scientific, Waltham, MA, United States) for 21 days. Besides, 1 × 10^4^ hAMSCs cultured on TCP with the same chondrogenic medium was considered as a control group for real time PCR.

#### 2.2.5 Safranin-O staining

Safranin-O (Merck KGaA, Darmstadt, Germany) identified the presence of cartilaginous proteoglycans in active chondrocytes. First, specimens were placed on a slide, then they were deparaffinized and washed with distilled water. After that, they were rinsed in Weigert’s iron hematoxylin for 10 min and washed with running tap water for about 10 min. Next, the samples were stained with a fast green solution for 5 min, then washed with an acetic acid solution, and rinsed in 0.1% Safranin-O solution for 5 min. Finally, the specimens were dehydrated and cleared with 95% ethyl alcohol, 100% ethyl alcohol, and xylene for 2 min, and a resinous medium was used for fixation. The samples were subsequently observed under a microscope. The threshold and measure of the photos were adjusted and processed. Then, the amount of staining was characterized and analyzed with ImageJ software.

#### 2.2.6 Alcian blue staining

Alcian blue (Merck KGaA, Darmstadt, Germany) was used to detect sulfated proteoglycans in cartilage tissue. First, the deparaffinized samples with xylene substitute were hydrated in 100%, 95%, 70%, and 50% ethanol, respectively. After that, the samples were washed with di-H_2_O for 5 min and incubated in 3% acetic acid for 3 min. Next, they were stained with a 1% Alcian blue solution with a pH of 2.5 for 30–60 min. Afterward, they were rinsed with tap water for 2 min. The samples were fixed in the xylene substitute and observed under a microscope. The measure and threshold of the photos were adjusted and processed. Then, the amount of staining was specified and analyzed by ImageJ software.

#### 2.2.7 Immunofluorescence assay and DAPI staining

First, for the immunofluorescence assay, a series of processes including the deparaffinization of the paraffin segments, hydration in xylene and an ethanol series, post-fixation with 4% paraformaldehyde, and two washes in phosphate-buffered saline were conducted. In the next step, the samples were permeabilized by incubation with 0.1% (v/v) Triton-X 100 diluted in PBS for 5 min at room temperature. Subsequently, the samples were washed with PBS three times for 5 min and then incubated with 2% goat serum (Sigma-Aldrich, Steinheim, Germany) diluted in PBS for 20 min at 4°C to block the nonspecific binding of antibodies. Cells were then incubated with primary antibodies, including anti-collagen-type-2 and anti-ACAN (Santa Cruz Biotechnology), overnight at 4°C. After labeling, unbound primary antibodies were removed by rinsing samples three times with PBS for 5 min. The samples were subsequently immersed in AleaFluor-488 goat anti-rabbit or goat anti-mouse immunoglobulin G (Invitrogen) secondary antibodies for 1 h at 4°C. Finally, the samples were washed three times with PBS for 5 min, and the nuclei of cells were stained with diluted (1:1000) 4′,6-di-amidino-2-phenyl-indole (DAPI: Applichem, Darmstadt, Germany) for 20 min at 4°C and rinsed three times with PBS. Then the cells were observed using a fluorescence microscope (FV500, Olympus Fluoview, Japan). The measure and threshold of the photos were adjusted and processed. Then, the number of stained cells was specified by analyzing the particle option by ImageJ software.

#### 2.2.8 Gene expression

After 21 days of chondrogenic differentiation, quantitative real-time PCR (qRT-PCR) analysis was conducted to assess the gene expression of Aggrecan (ACAN) and collagen type II (COL2) as chondrogenic genes and Β-actin as a housekeeping gene. For these purposes, total RNA was extracted using an RNA extraction kit (RNeasy Mini Kit, Qiagen, United States), and cDNA synthesis was performed with the Revert Aid first-strand cDNA synthesis kit. qRT-PCR reactions were carried out *via* the following cycles: 95°C for 3 min as an initial denaturation step, followed by 45 cycles of denaturation at 95°C for 30 s, annealing at 60°C for 30 s, and extension at 72°C for 40 cycles for 30 s. Primers were designed using Primer-BLAST online software from the National Center for Biotechnology, and their specificity was investigated by BLAST (NCBI). The primer sequences are demonstrated in Table 1. The melting curve analysis was attached at the end of the amplification procedure, and it indicated no nonspecific amplification. The relative changes in target gene expression were quantified using the ΔΔCt method.

**TABLE 1:** Primers sequence e for quantitative real-time PCR.

Gene	Forward sequence	Revers sequence
*β* *-actin*	5-AGC​ACA​GAG​CCT​CGC​CTT-3	5-CAC​GAT​GGA​GGG​GAA​GAC-3
*ACAN*	5-CCA​CCA​CCT​ACA​AAC​GCA​GA-3	5-GAT​TTG​GAG​GGG​TGA​GTG​GG-3
*Col 2*	5-TCT​ACC​CCA​ATC​CAG​CAA​AC-3	5-GCG​TAG​GAA​GGT​TCA​TCT​GGA-3

#### 2.2.9 Statistical analysis

All experiments were performed with n = 3. Statistical analyses of the results were carried out by GraphPad, Prism software (V.9, United States) using one-way and two-way ANOVA and also, Tukey’s multiple comparisons tests were used for means. The swelling ratio, degradation test, MTT assay, quantification of alcian blue staining, quantification of safranin O staining, quantification of immunofluorescence staining and gene expression were analyzed *via* two-way ANOVA, and Porosity Measurement was analyzed via one-way ANOVA. The possibility values less than 0.05 (*p*-value <0.05) were considered significant.

## 3 Results

### 3.1 FTIR test

**FIGURE 1:**
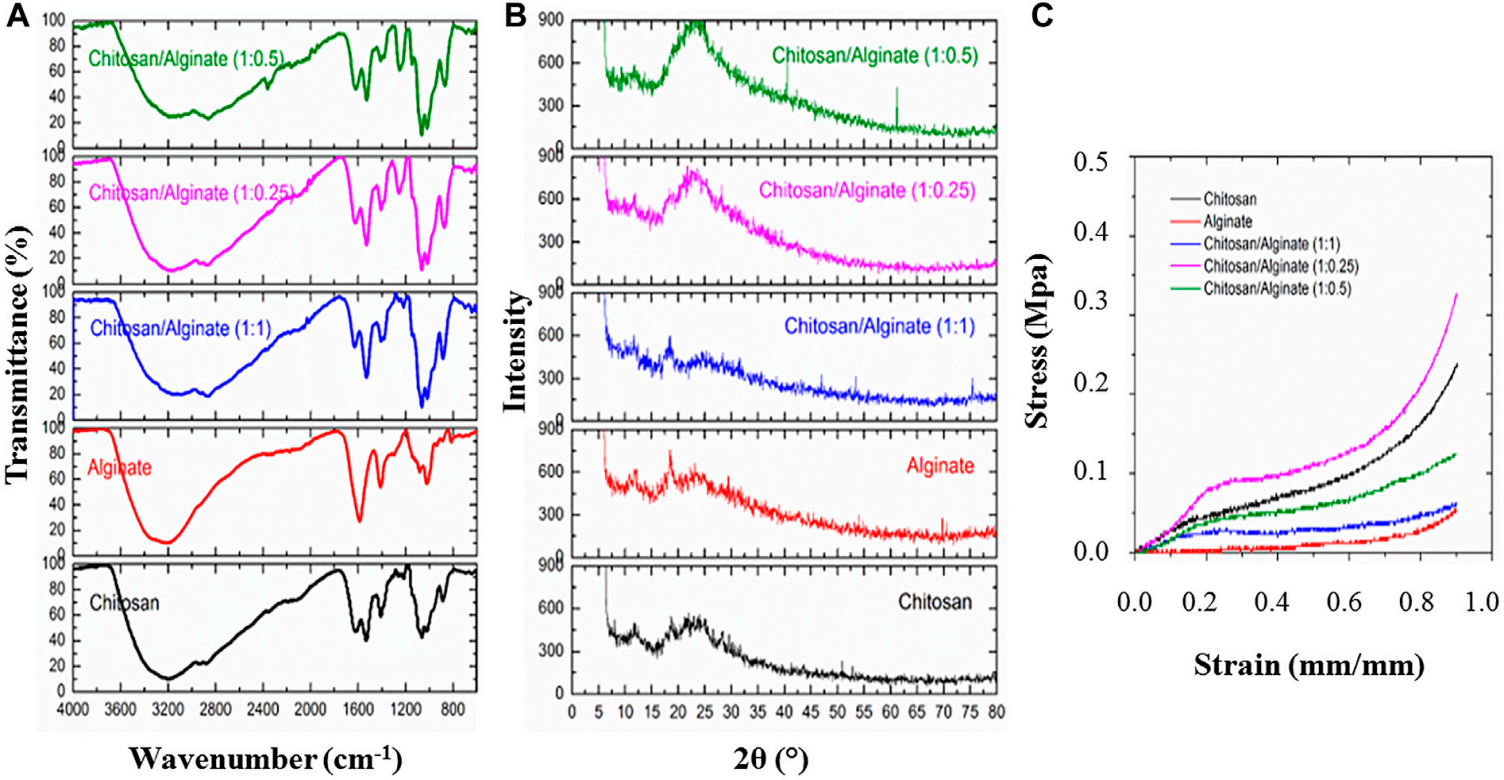
**(A)** ATR-FTIR spectra of Chi, Alg, Chi/Alg 1:0.25, Chi/Alg 1:0.5, Chi/Alg 1:1 scaffolds, **(B)** The X-ray diffraction (XRD) spectra of Chi, Alg, Chi/Alg 1:0.25, Chi/Alg 1:0.5, Chi/Alg 1:1 scaffolds, **(C)** Stress−strain curves of freeze-dried hydrogels.

The results of the infrared spectroscopy test for pure chitosan, pure alginate, and their alloys are presented in Figure 1A. Pure chitosan has characteristic absorption peaks related to carbonyl groups (C=O) at 1620 cm^-1^ and amine (NH_2_) at 1531 cm^-1^. Furthermore, the asymmetric and symmetric stretches of carbonyl groups at 1620 cm^-1^ and 1409 cm^-1^ are related to the residues of type I amide groups in the structure of chitosan and originate from the structure of chitin (the basic constituent of chitosan). Type I amine has an index absorption in the range of 3400–3500 cm^-1^ due to the asymmetric and symmetric stretching of NH groups. However, due to the coincidence of these stretching vibrations with the presence of hydroxyl (OH) groups and its characteristic broad peak at about 3205 cm^-1^, these cases have been merged. In addition, the double peaks in the range of 2800–3000 cm^-1^ belong to the stretching vibrations of methylene groups (CH) in the structure of chitosan such as chitosan rings. The absorption peaks in the range of 1000–1200 cm^-1^ originate from the saccharide structure in chitosan. The absorptions around 1400, 1300, and 1150 cm^-1^ are respectively due to stretching movements of C-O group, in-plane bending of OH, and C-O-C groups (glyosidic linkages between chitosan units). The peaks at 1065 and 1020 cm^-1^ belong to C-OH stretching and C-N vibrations, and absorptions at 888 and 646 cm^-1^ are related to C-C stretching vibrations and wagging vibrations of NH groups. Besides, the bending vibrations of the chitosan ring occur at around 600 cm^-1^.

The spectrum of pure alginate (sodium alginate) has a broad peak in the range of 3000–3500 cm^-1^ centered at 3207 cm^-1^ due to the stretching movements of hydroxyl groups. Moreover, the double peaks in the range of 2800–3000 cm^-1^, which are mainly observed as shoulders, are related to the stretching vibrations of methylene groups (CH) in the alginate structure, such as alginate rings. The absorption peak of 1588 and 1412 cm^-1^ confirms the asymmetric and symmetric stretching movements of CO_2-_ ion groups. The absorption peak in the range of 1300 cm^-1^ indicates the skeletal vibrations of the alginate structure. The peaks of 1080–1023 cm^-1^ also indicate the asymmetric stretching movements of C-O-C groups in the chemical structure of alginate.

Due to mixing alginate with chitosan, we can expect chemical (amide formation) and physical interactions between the carboxylic acid groups of alginate and the amine and amide groups of chitosan, respectively. In this regard, in comparing the spectrum of chitosan and alginate mixtures with the spectrum of pure substances, the absorption peak of 1588 cm^-1^ indicates the removal of acidic CO_2-_ groups in the alginate spectrum; on the other hand, the absorption peak at 1620 cm^-1^ confirming the removal of carbonyl groups in the chitosan spectrum changes in the spectrum of the mixtures. According to the previous information, this result, along with the simultaneous large shift in the center of the broad peak originating from the hydroxyl and amine groups around 3205 and 3207 cm^-1^ for chitosan and alginate to smaller wavenumbers for the mixtures, confirms the aforementioned interactions between these two substances (Figure 1A).

### 3.2 XRD test

The results of the X-ray diffraction spectroscopy test for pure chitosan, pure alginate, and their alloys are given in Figure 1B. In the spectrum of pure chitosan, the characteristic peak in the 12° range indicates type I crystal, which originates from the hydrated crystal structures in chitosan. Note that chitosan has at least 5% water bound to the structure even if it is extremely dried. The second characteristic peak in the 23° range shows regular chitosan structure crystals (type II crystals). This second characteristic peak indicates the high degree of crystallinity in chitosan structure. Due to the similarity of the structure, alginate also has the above-mentioned characteristic peaks, suggesting its semi-crystalline structure. Evidently, by adding alginate to chitosan, the spatial structure and shape of the crystals do not change due to the non-shifting of the characteristic peaks; however, at low to medium degrees of alginate loading, the characteristic peaks of crystallinity become more pronounced, which can show the strengthening of crystallinity due to the synergism of the materials. However, at high alginate loading (1:1 ratio), the intensity of the characteristic peaks and this synergism is reduced. This phenomenon can affect the physical and mechanical properties of the system (Figure 1B).

### 3.3 Compression test

The mechanical strength of the samples was obtained through the compressive strength test. The stress-strain diagram resulting from the compressive loading test on the samples is displayed in Figure 1C. The mechanical characteristics of the samples are extracted from the above graphs and given in Table 2. Pure chitosan shows higher mechanical resistance than pure alginate at any desired strain. In particular, based on Table 2, the elastic modulus of the linear loading area and the yield stress of alginate are much smaller than those of pure chitosan. This can be rooted in the lower intensity of physical interactions between chains, lower crystallinity, and greater mobility of alginate chains compared to chitosan. By adding alginate to chitosan up to a ratio of 1:0.25, the mechanical strength of the mixture increases in terms of elastic modulus, yield stress, and yield strain limit, and then declines. According to the results of the infrared spectroscopy and X-ray diffraction test, the formation of chemical and physical interactions between chitosan and alginate chains, along with the synergy of alginate chains on the crystallinity of chitosan chains, enhanced the mechanical strength of the mixture. In this regard, the degree of deacetylation of chitosan and the number of amino groups on chitosan chains should be taken into account when forming the aforementioned interactions with alginate, which has proved effective at this mixing ratio. However, with a further rise in the ratio of alginate in the mixture, the effective number of interactions between the chains and, especially, the intensity of crystallinity of the system declined, thereby reducing the mechanical properties. In this regard, the distribution level of the alginate phase in chitosan is another effective parameter for improving mechanical properties, which can be greatly enhanced at the ratio of Chi/Alg 1:0.25 (Figure 1C).

**TABLE 2:** Mechanical characterization of 3D-Printed scaffolds.

Sample	Modulus (kPa)	Yield stress (kPa)	*r* ^2^	Yield strain
Chitosan	269	43	0.973	0.168
Alginate	13	4	-	0.400
Chitosan/Alginate 1:0.25	355	78	0.965	0.200
Chitosan/Alginate 1:0.5	183	39	0.944	0.200
Chitosan/Alginate 1:1	156	23	0.947	0.165

### 3.4 Scanning electron microscopy

**FIGURE 2:**
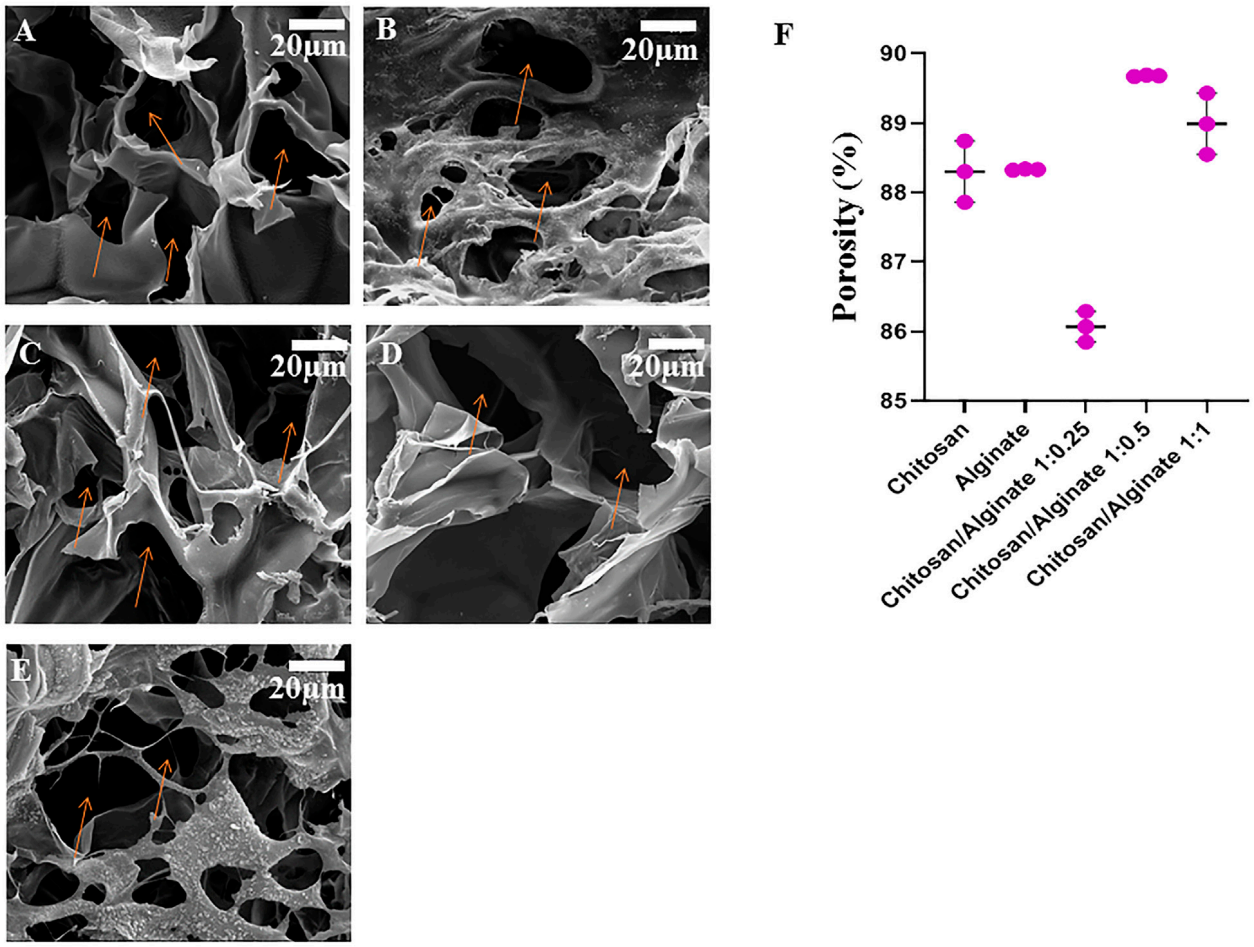
Morphology of freeze-dried hydrogel scaffolds by scanning electron microscopy (The orange arrows show the pore of scaffolds). **(A)** chitosan scaffolds, **(B)** alginate scaffold, **(C)** Chi/Alg 1:0.25, **(D)** Chi/Alg 1:0.5, **(E)** Chi/Alg 1:1 and **(F)** the porosity changes of the freeze-dried hydrogels (n = 3).

The microscopic surface specifications and macroscopic features of five different freeze-dried hydrogels were observed and shown in Figure 2. As shown in Figures 2A–E, the Chi, Alg, and Chi/Alg freeze-dried hydrogels almost have a homogeneous distribution of white color, which demonstrates the uniform scattering of Chi, Alg, or both.

Two critical factors for cartilage repair are the pore structure and porosity of the scaffolds because they have a notable effect on the exudates and blood attraction.

The pore structure and pore size of the Chi, Alg, Chi/Alg 1:0.25, Chi/Alg 1:0.5, and CS/Alg 1:1 hydrogel were evaluated using SEM (Figure 2). All scaffolds have a three-dimensional structure with interconnected pore construction and irregular formation.

According to the SEM images (Figure 2) and quantitative data (Figure 2F), the weight ratio of Chi and Alg significantly depends on the pore size of the composite scaffolds; the higher the mass ratio of Chi and Alg, the smaller the pore size of these hydrogels. Interaction between negatively charged anionic Alg and positively charged Chi might be the cause of these results. Furthermore, the ethanol displacement method was used to measure the porosity of the manufactured scaffolds. Figure 2F depicts the porosity of different scaffolds. The porosity in Chi/Alg 1:0.5 is higher than that of other scaffolds, but statistical analysis determined that this difference was no significant.

### 3.5 Swelling

**FIGURE 3:**
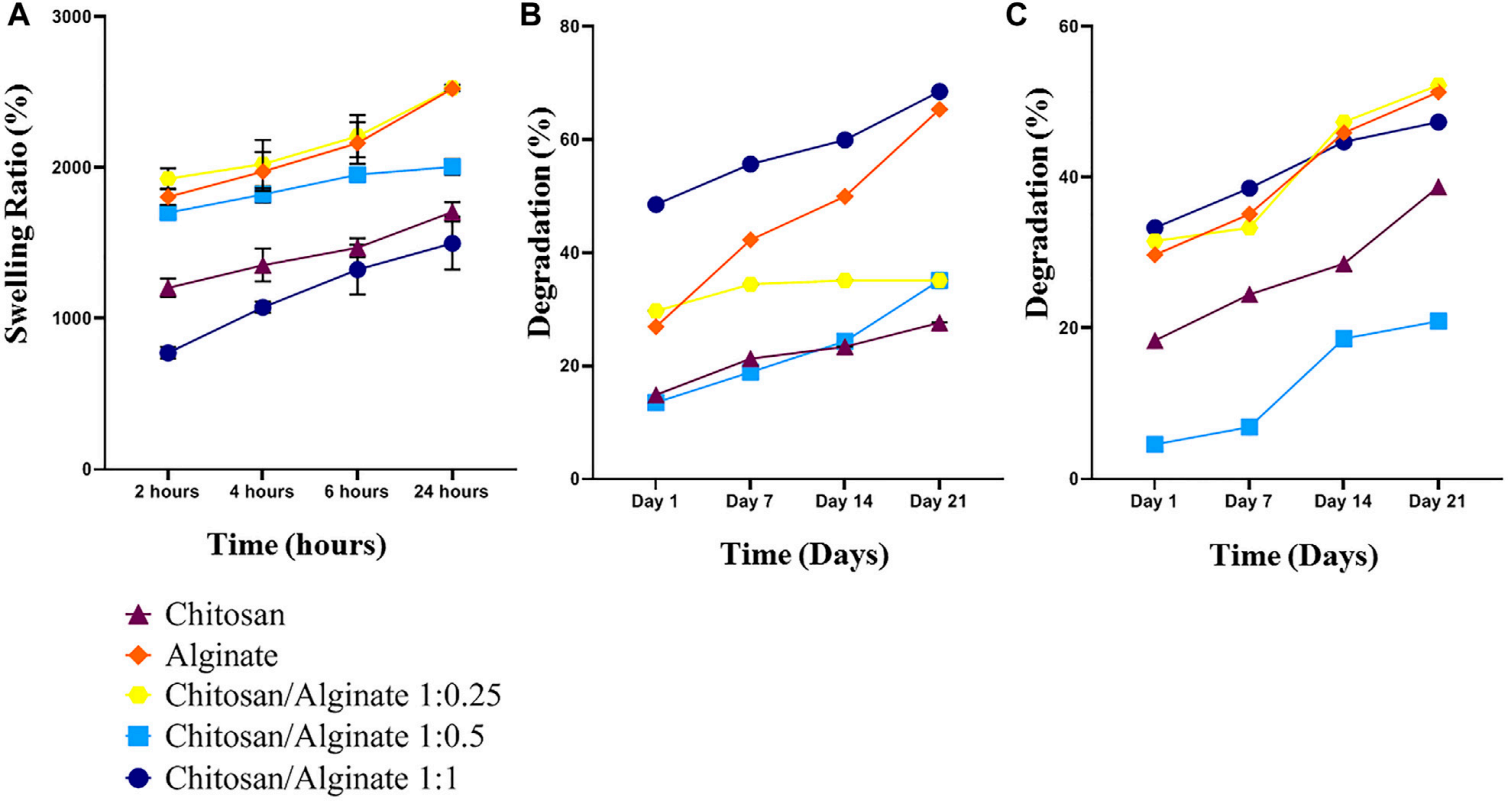
**(A)** Swelling behavior of the freeze-dried hydrogel scaffolds. **(B,C)** Weight loss of Chi, Alg, Chi/Alg 1:0.25, Chi/Alg 1:0.5, Chi/Alg 1:1 scaffolds during soaking in PBS and PBS-Lipase for 28 days (n = 3 in each group, *p ˂ 0.05).

The swelling behavior of the hydrogels in phosphate-buffered saline (PBS) solution can be used to evaluate the water uptake capability of the scaffolds. To measure the water uptake and retention capacity of Chi, Alg, Chi/Alg 1:0.25, Chi/Alg 1:0.5, and Chi/Alg 1:1, they were immersed in 1X PBS solution (Figure 3A). The results demonstrated the differences in their swelling behavior, and the water uptake capacity of the Alg and Chi/Alg 1; 0.25 scaffolds were shown to be higher when compared to the other scaffolds. It was previously reported that alginate can quickly absorb water and maintain 200–300 times its own mass of water. The increase of surface in scaffold swelling can enhance cell adhesion and infiltration.

### 3.6 Degradation

Some crucial parameters in cartilage tissue engineering are *in vitro* biodegradation and *in vitro* degradation, which should be taken into consideration. Tissue growth and matrix deposition need space and scaffold biodegradation prepares this space for tissue construction. In the present study, both degradation and biodegradation were evaluated, and the results demonstrated that pure Alg scaffolds have high degradation in both groups. In the group with an enzyme in PBS, the highest degradation belongs to Chi/Alg 1:0.25, and in the group without an enzyme, it belongs to Chi/Alg 1:1 (Figures 3B, C).

### 3.7 MTT assay

**FIGURE 4:**
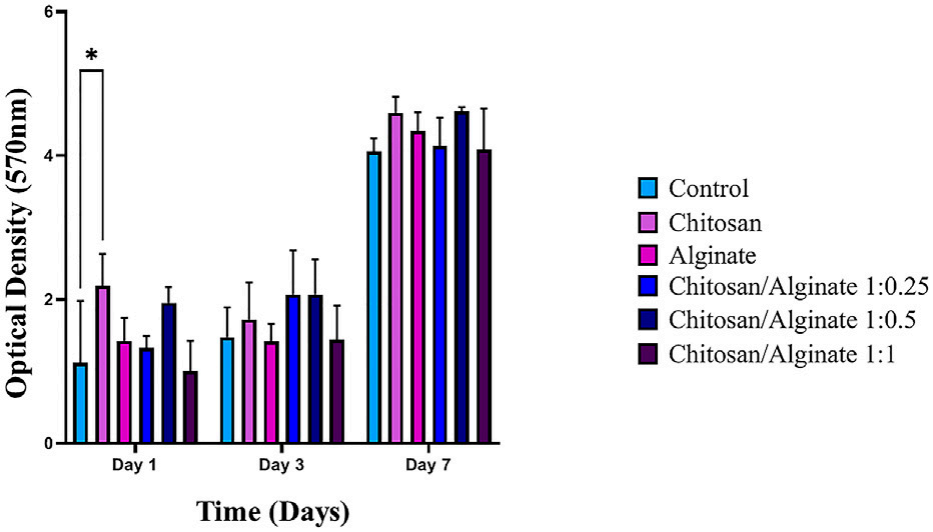
The determined proliferation rate of hADSCs on Chi, Alg, Chi/Alg 1:0.25, Chi/Alg 1:0.5, Chi/Alg 1:1 scaffolds and TCP by MTT assay after 1,3,7 and 14 days after cell culture. (n = 3, *p ˂ 0.05).

Toxicity and biocompatibility of fabricated scaffolds are among the significant issues in tissue engineering. One of the suitable assays for measuring the cytotoxicity of scaffolds is the MTT assay. This assay is based on the capability of cellular mitochondrial dehydrogenase to decrease the yellow-colored tetrazolium salt to purple-colored formazan crystals. MSCs were applied in this study to evaluate the toxicity level of the fabricated scaffolds. Cell viability on the prepared scaffolds (Chi, Alg, Chi/Alg 1:0.25, Chi/Alg 1:0.5, and Chi/Alg 1:1) at different time intervals is shown in Figure 4. The fabricated scaffolds demonstrated biocompatibility and non-cytotoxicity. There was no difference in the viability and cell proliferation between these five scaffold groups after different days. Biocompatibility of all the scaffolds increased over time, but they showed no significant differences from each other in terms of biocompatibility.

### 3.8 Safranin O and alcian blue staining

**FIGURE 5:**
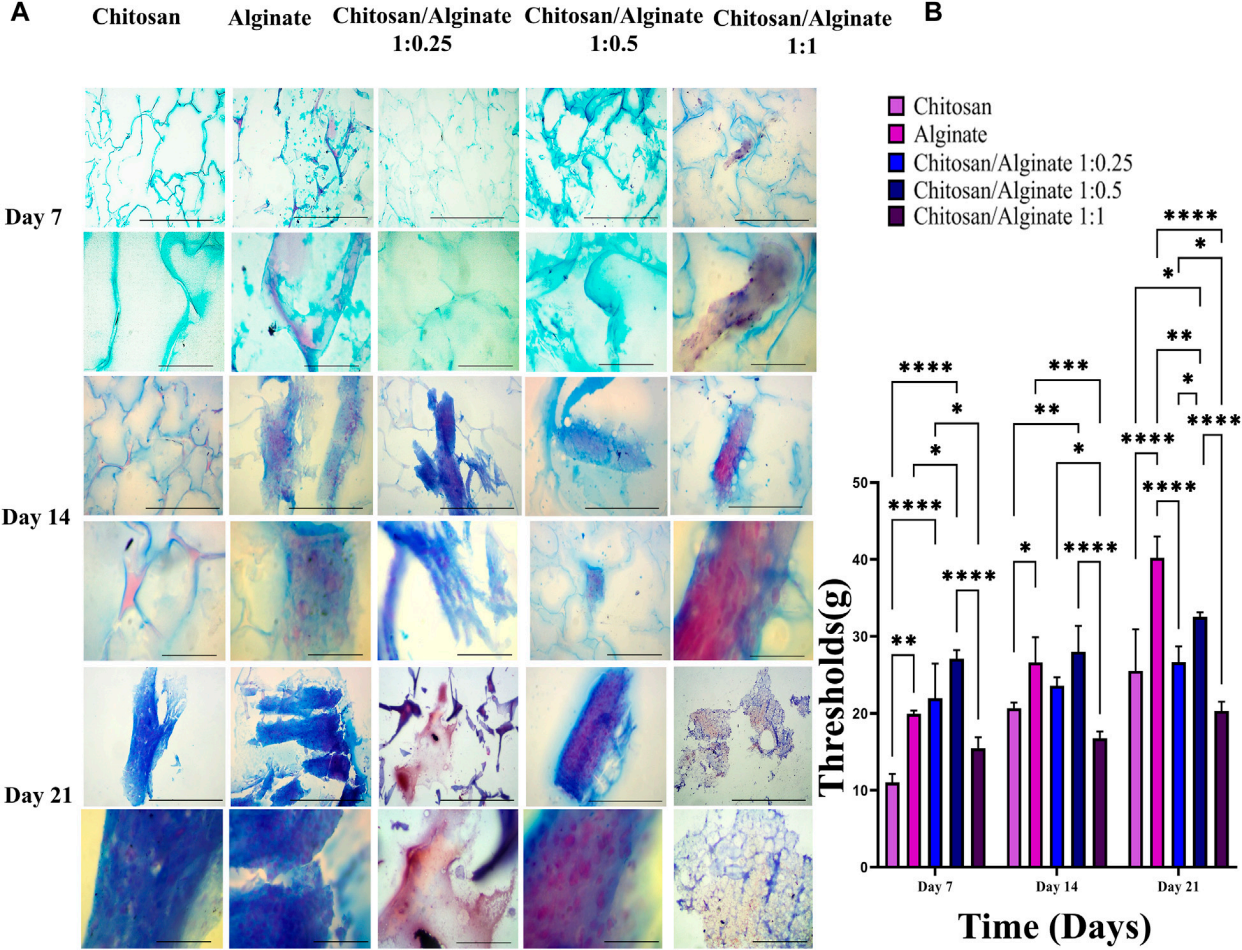
**(A)** Histological analysis including alcian blue staining in day 7, 14 and 21 after chondrogenic differentiation in hADSCs cultured on Chi, Alg, Chi/Alg 1:0.25, Chi/Alg 1:0.5, Chi/Alg 1:1 hydrogels. In each day the bars of first row represent 100 µm and the bars of second row represent 20 µm **(B)** Quantification of alcian blue staining (mean ± SD; *p ˂ 0.05, **p ˂ 0.01, ***p ˂ 0.001, ****p ˂ 0.0001).

**FIGURE 6:**
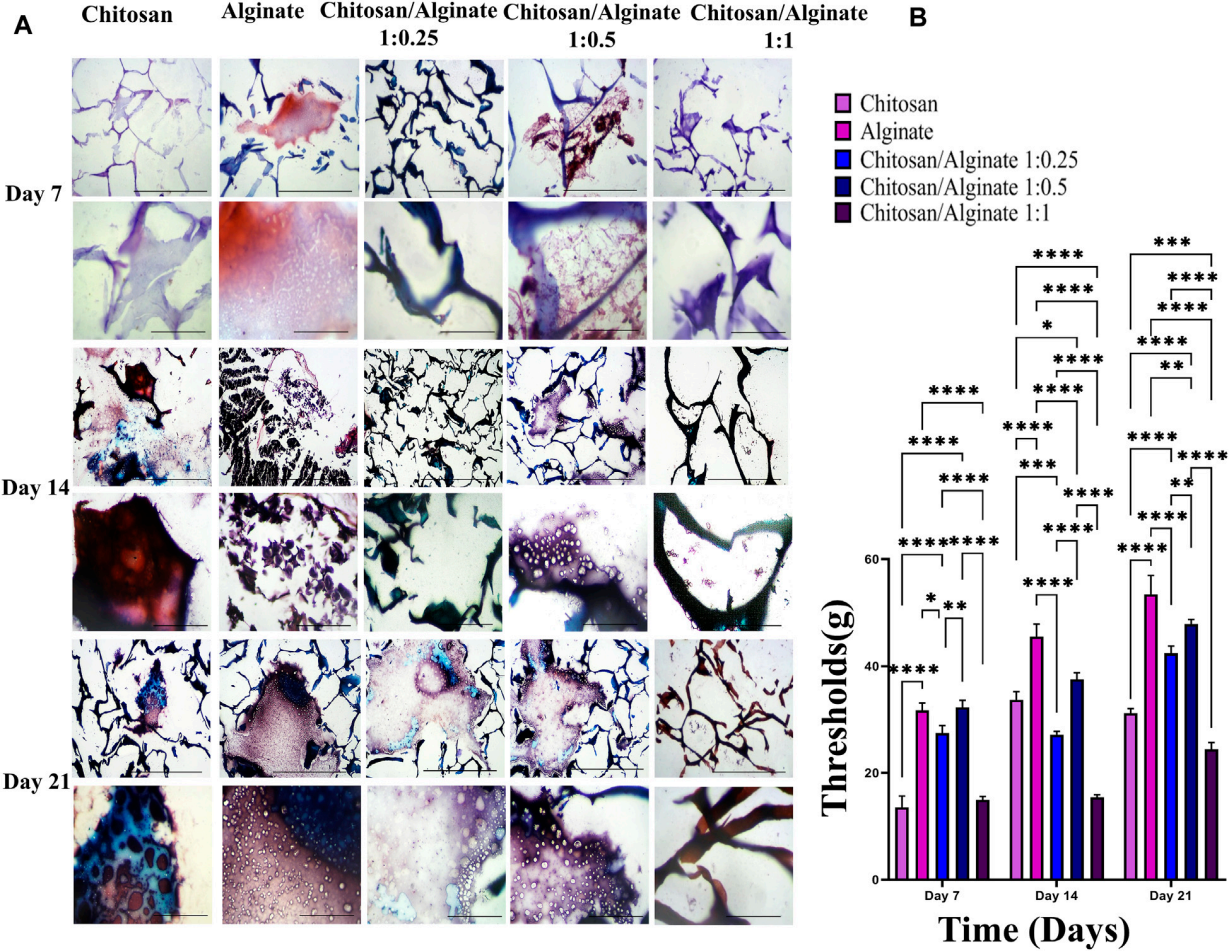
**(A)** Histological analysis including safranin O stainig in day 7, 14 and 21 after chondrogenic differentiation in hADSCs cultured on Chi, Alg, Chi/Alg 1:0.25, Chi/Alg 1:0.5, Chi/Alg 1:1 hydrogels. In each day the bars of first row represent 100 µm and the bars of second row represent 20 µm **(B)** Quantification of safranin O staining (mean ± SD; *p ˂ 0.05, **p ˂ 0.01, ***p ˂ 0.001, ****p ˂ 0.0001).

Safranin O (Figures 6A, B) and alcian blue (Figures 5A, B) staining indicate proteoglycans in active chondrocytes. In the present study, staining was performed 7, 14, and 21 days after the cell culture and differentiation. Proteoglycan production was demonstrated in all five groups, including Chi, Alg, Chi/Alg 1:025/Chi/Alg 1:0.5, and Chi/Alg 1:1, which suggests that cell differentiation and chondrocyte production were conducted successfully. In qualitative comparison, the proteoglycan production increased in all groups on day 21 compared to day 14 and on day 14 compared to day 7. Chi/Alg 1:0.5 has highest threshold on day 7 and 14 after cell differentiation in alcian blue staining and also has highest threshold on day 7 in safranin O staining. Alginate has highest threshold on day 21 after cell differentiation in alcian blue staining and also has highest threshold on day 14 and 21 in safranin O staining. Enhanced production of proteoglycans was caused by the induction of cell growth, adherence, and differentiation (Figure 5; Figure 6).

### 3.9 Immunofluorescence assay

**FIGURE 7:**
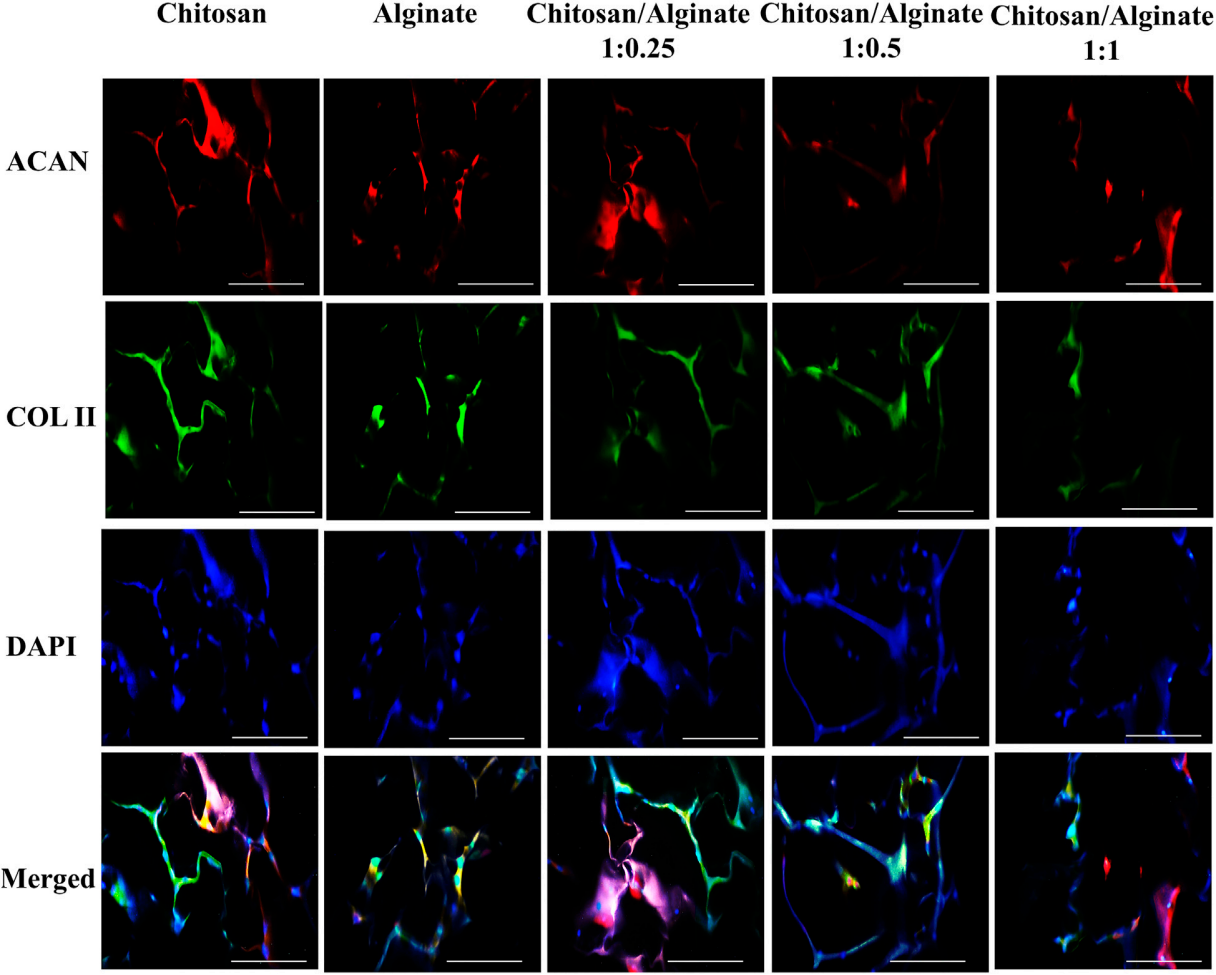
DAPI staining and immunofluorescence staining of chondrogenic markers include COL II and ACAN in cultured hADMSCs on Chi, Alg, Chi/Alg 1:0.25, Chi/Alg 1:0.5, Chi/Alg 1:1 scaffolds after 21 days induction in a chonrogenic differentiation medium. Bars represent 50 µm.

**FIGURE 8:**
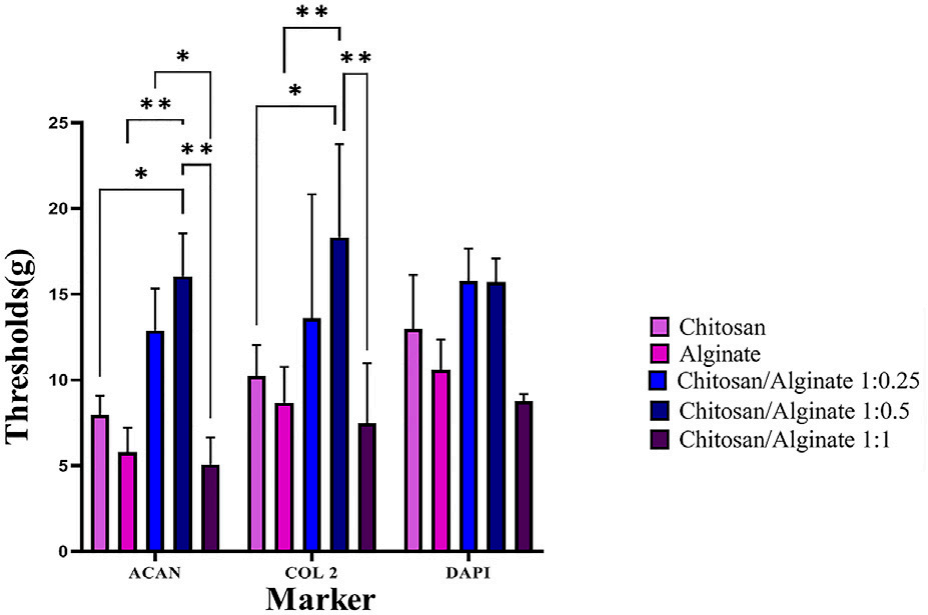
Quantification of immunofluorescence staining. (mean ± SD; *p ˂ 0.05, **p ˂ 0.01).

The expression of *COL 2* and *ACAN,* as chondrocyte-specific protein markers, was evaluated using the immunofluorescence staining 21 days after cell differentiation (Figures 7, 8). Immunofluorescence staining image demonstrated the expression of COL 2 and ACAN in all groups, including pure Chi, pure Alg, Chi/Alg 1:0.25, Chi/Alg 1:0.5, and Chi/Alg 1:1 composite hydrogels. The expression level of the aforementioned markers was observed in all three composite groups, including Chi/Alg 1:0.25, Chi/Alg 1:0.5, and Chi/Alg 1:1; however, Chi/Alg 1:0.5 and Chi/Alg 1:0.25 hydrogels showed higher expression than Chi/Alg 1:1 and Chi/Alg 1:0.5 hydrogel showed higher expression than Chi/Alg 1:0.25 hydrogel, which might be due to the degradation rate of Chi/Alg 1:1 hydrogel (Figure 7; Figure 8).

### 3.10 Real-time RT-PCR

**FIGURE 9:**
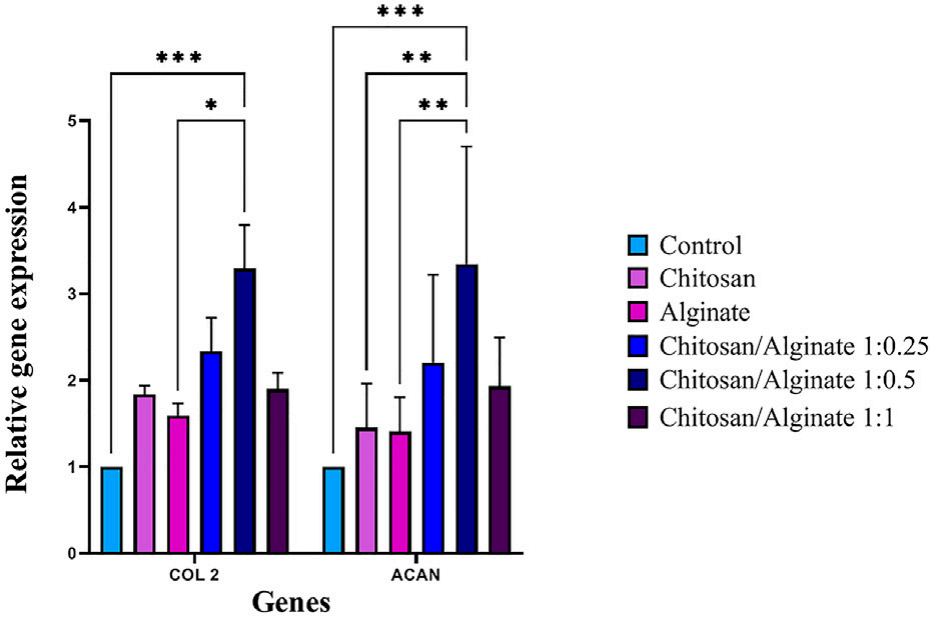
Relative expression of COL II and ACAN in hAD-MSCs on Chi, Alg, Chi/Alg 1:0.25, Chi/Alg 1:0.5, Chi/Alg 1:1 scaffolds, and TCPs during the chondrogenic process genes after 21 days (n = 3, *p ˂ 0.05, **p ˂ 0.01, ***p ˂ 0.001).

Immunofluorescence staining qualitatively demonstrated the protein expression of COL 2 and ACAN, whereas real-time PCR quantitatively evaluated COL 2 and ACAN gene expressions. COL 2 and ACAN *were expressed in the c*ontrol and five experimental groups, but their expression rate was varied. Chi/Alg 1:0.5 hydrogel had a higher expression of these genes compared to the other groups. The increased density of alginate in Chi/Alg 1:0.5 hydrogel in comparison to Chi/Alg 1:0.25 hydrogel and the lower degradation rate of Chi/Alg 1:0.5 hydrogel in comparison to Chi/Alg 1:1 hydrogel might be the reasons for the induced expression of chondrocyte-specific genes (Figure 9).

## 4 Discussion

Cartilage is a highly specialized connective tissue that has a limited ability for self-repair after injury due to avascularity and a low supply of repair cells (Zhang et al., 2009; Zhang X. et al., 2023). To treat cartilage deficiencies, numerous surgical techniques exist, but their efficiency has not yet been proven. Tissue engineering with a combination of biocompatible hydrogels, cells, and bioactive molecules provides an alternative approach (Liu et al., 2017). Hydrogels have elastic networks with a high water content that can simulate hydrated natural cartilage. Moreover, the injectability and adjustable mechanical and biochemical properties of hydrogels make them ideal scaffolds for cartilage tissue (Wu et al., 2021). Scaffolds encourage the growth, proliferation, and differentiation of cells (Hu et al., 2021). Scaffolds mimic the extracellular matrix (ECM) and make a proper porosity for cell attachment and growth (Shariati et al., 2022). Biodegradability, biocompatibility, and non-toxicity are the properties of suitable scaffolds (Ebrahimi et al., 2022). As it mentioned a suitable tissue-engineering scaffold should have proper porosity and interconnected pore with adequate size. The SEM images of 5 hydrogels are depicted in Figure 2 and the porosity of our all groups is above 80%. These pores supply a specified microenvironment for promoting cell migration and vascularization, transport of nutrients and gases, and removal of waste materials. In addition, these structures can withstand external loading stresses (Hassani et al., 2022).

The SEM image demonstrated a porous structure with a rough surface for the hydrogel, which is proper for cartilage tissue engineering. The pores’ diameter was in the range of 38–92 μm, calculated via ImageJ. This pore size range is adequate for cell attachment, and it is proper pore size range for cartilage tissue engineering (Ramzan et al., 2022). Higher porosity and large pores inside the scaffolds explain the greater permeability and speedy degradation (Hassani et al., 2022). Therefore, the degradation of cross-linked hydrogels by calcium including Alg and Chi/Alg 1:1 scaffolds is almost the fastest among the groups. However, it is more related to the presence of alginate and its percentage than to the crosslink factor. As also confirmed by our results, Alg and Chi/Alg 1:1 hydrogels have the highest degradation rate and biodegradation rate and Chi/Alg 1:0.5 hydrogel has the lowest degradation rate and biodegradation rate (Figure 2B, C). MTT results showed in Figure 4 and demonstrated that the cell growth in all groups increased from day 1 to day 7. Increase cell growth showed our scaffolds are biocompatible and nontoxic. Mostafa Saberian et al. (2021) worked on Fabrication and characterization of alginate/chitosan

Hydrogel combined with honey and aloe vera for wound dressing applications. Their MTT results showed Chi/Alg scaffolds are biocompatible in 1,3 and 7 days post cell culture and our results showed Chi/Alg scaffolds biocompatibility too(Saberian et al., 2021).

Due to its biocompatibility, chitosan is currently utilized in tissue engineering (Ahsan et al., 2018). It is a deacetylated chitin with the chemical formula (C_6_H_11_O_4_N)n. Chitosan free amino groups can bond to several molecules like and metal ions, proteins, fat, DNA, RNA, (Rezaei et al., 2021), and it is compatible with other materials such as metals, polymers, and proteins to make bio-functional composite materials (Zhang J. et al., 2023). Chitosan is a cost-effective natural biopolymer, which derived from reproducible and natural source. The most popular exclusivity of chitosan making it suitable for cartilage tissue engineering is its similarities with N-glycosaminoglycans, which are important compounds of connective tissues (Vukajlovic et al., 2019; Becerra et al., 2022; Sharifianjazi et al., 2022).

Alginate is a poly-anionic copolymer containing homopolymeric blocks of (1,4)-linked-β-D-mannuronate (M) and α-L-guluronate (G) residues (Wang et al., 2003; Catanzano et al., 2015). It is a highly hydrophilic carbohydrate, derived from brown sea algae and bacterial sources such as *Azotobacter* and *Pseudomonas*. A determining factor to trap cells, DNA, and protein is a formed structure. Some properties of alginate include non-immunogenicity, chemical adaptation, affordability, low toxicity, biodegradability, biocompatibility, unique water attraction, and significant crosslinking ability; all this makes it a suitable candidate for medical application and tissue engineering (Azari et al., 2022; Soleimanpour et al., 2022).

In this study, FTIR and XRD were performed to identify chitosan and alginate ingredients. The functional groups of organic and inorganic combinations and the intermolecular interaction among different ingredients were identified *via* FT-IR. Jayachandran Venkatesan et al. (2014) worked on a chitosan-alginate biocomposite containing fucoidan for bone tissue engineering. Their FTIR results showed an intense peak at 1613 cm^-1^. They noted that this peak belongs to the superposition of the bands specified to the carboxylate group of alginate and the amine group of chitosan, and the lower stretching frequency in OH detected from 3433 cm^-1^ to 3420 cm^-1^ indicates the existence of intermolecular hydrogen bonds in the chitosan-alginate system (Venkatesan et al., 2014).

As mentioned before, chitosan and alginate are natural polymers, with hydrogel-forming ability, which contain hydrophilic polymer networks and attract a large amount of water. The swelling ratio shows the hydrogels’ water absorption ability. Based on the results, the lowest swelling ratio belonged to Chi/Alg 1:1 and the highest swelling ratio belonged to pure Alg and Chi/Alg 1:0.25. As Alg has unique water absorption, the reason for the decreasing swelling ratio with increasing Alg ratio could be the enhanced degradation in Chi/Alg 1:1. Figures 3B, C show that the highest degradation rate belonged to pure Alg and Chi/Alg 1; 1. When a hydrogel is submerged in water, it swells until the osmotic powers that aid to develop the polymer network are equilibrated by the elastic powers from the stretched parts of the polymer (Ramzan et al., 2022).

The combination and organization of the extracellular matrix (ECM), especially the pericellular matrix (PCM), in cartilage is vital to its biomechanical performance; the existence of entrapped proteoglycans such as aggrecan in type II collagen fibrillar network creates mechanical resilience underweight-bearing (Gilbert et al., 2021).

ECM is formed of a pericellular, territorial, and interterritorial matrix (Guilak et al., 2018). A chondron-surrounding structure including of a high condensation of soluble proteoglycans (PGs) with fast turnover, included in a condensed meshwork of fibrous proteins with low turnover is territorial matrix (Caron et al., 2012). PGs employed as a cell cushion. Core protein of PGs restricted by long chains of starch-like molecules named glycosaminoglycans (GAGs), which can be classification of PGs includes large, predominant PGs, like aggrecan, and small, minor PGs, like decorin, biglycan, asporin, lumican, and fibromodulin. GAGs include hyaluronic acid (HA), dermatan sulfate, chondroitin sulfate, heparan sulfate, and keratan sulfate, while the major GAGs connected to the core protein contain chondroitin-4/6-sulfate and keratan sulfate (Liu et al., 2022).

Collagen is the main fiber in ECM (75% of the dry weight) (Carballo et al., 2017), being the most important constituent to provide tensile strength (Becerra et al., 2010).

A high GAG content yields low hydraulic permeability and high swelling pressure properties to the tissue, which are critical for the load-bearing requirements of joints (Uzieliene et al., 2023).

As mentioned, safranin O staining, alcian blue staining, real-time PCR, and immunofluorescence assay were performed to demonstrate the differentiation process. Safranin O and alcian blue staining showed proteoglycans and GAGs. Sandra Escalante et al. (2022) worked on chemically crosslinked hyaluronic acid-chitosan hydrogel for application on cartilage regeneration and Dechao Yuan et al. (2015) worked on cartilage tissue engineering using combination of chitosan hydrogel and mesenchymal stem cell. Sandra Escalante et al. (2022) used of safranin O staining and alcian blue staining for showing production of proteoglycans and Dechao Yuan et al. (2015) used of safranin O staining for demonstrating of proteoglycans production. In safranin O staining red to purple color and in alcian blue staining blue color demonstrate the proteoglycans production (Yuan et al., 2015; Escalante et al., 2022).

As shown in Figure 5 and Figure 6, the highest amount of proteoglycans and GAGs is in Alg and Chi/Alg 1:0.5 groups. Real-time PCR was performed to identify the expression level of *COL 2* and ACAN *genes, and* immunofluorescence assay *was* also carried out *to indicate their protein expression on day 21 post-differentiation. Genes expression was observed in all the groups, and the highest expression belonged to the Chi/Alg 1:0.5 group.*

Elke Gossla et al. (2021) worked on Anisotropic Chitosan Scaffolds Generated by Electrostatic.

Flocking Combined with Alginate Hydrogel Support Chondrogenic Differentiation. They measured *COL2*, *ACAN* and *COL1*gene expression on day 21 for demonstration of chondrogenic differentiation. In their research *COL2* gene expression was higher in Chi/Alg group than other groups but *ACAN* gene expression was higher in Alg group than others (Gossla et al., 2021). However, in our work the gene expression of both *COL2* and *ACAN* were higher in Chi/Alg groups (especially in Chi/Alg 1:0.5) than pure Chi and pure Alg.

Xiaodie Zhang et al. (2023) worked on Comparative study of alginate and type I collagen as biomaterials for cartilage stem/progenitor cells to construct tissue-engineered cartilage *in vivo*. They used of immunofluorescence staining for showing the COL2 formation to prove chondrogenic differentiation (Zhang X. et al., 2023). However, we used of immunofluorescence staining for demonstrating COL2 and ACAN as tow important chondrogenic factor.

Immunofluorescence staining showed protein qualitative expression in all the groups. The results of real time PCR and immunofluorescence staining are match together and both of them demonstrated highest gene and protein expression in Chi/Alg 1:0.5 group.

## 5 Conclusion

In this study, five different types of hydrogel scaffolds were prepared for use in cartilage tissue engineering. Chemical ionic interactions, mechanical properties, biological activity and differentiation properties were observed between chitosan and alginate, which led to the improved bioactivity of the scaffolds. The combining of Chi-Alg with different ratios showed better activity than one of them alone in the regeneration of cartilage tissue. According to the results obtained in the present study, it can be concluded that the Chi-Alg-scaffolds would be a great promising biomaterial to repair cartilage lesions in osteoarthritis patients.

## Data Availability

The raw data supporting the conclusion of this article will be made available by the authors, without undue reservation.

## Abbreviations

ACAN, aggrecan; Alg, alginate; ATR-FTIR, Attenuated Total Reflectance Fourier-Transform Infrared; Chi, chitosan; COL2, collagen type II; DAPI, 4′,6-Diamidino-2-phenylindole dihydrochloride; diH2O, deionized water; DMEM, Dulbecco’s Modified Eagle’s Medium; DMSO, Dimethyl sulfoxide; ECM, Extracellular matrix; FBS, Fetal bovine serum; H, Hour; hAMSCs, human Adipose tissue-derived Mesenchymal Stem Cells; MTT, (3-(4,5-Dimethylthiazol-2-yl)-2,5-diphenyl Tetrazolium Bromide); OD, Optical density; PBS, Phosphate-buffered saline; Penstrep, penicillin and streptomycin; SEM, Scanning Electron Microscopy; TCP, Tissue culture plastic; TE, Tissue engineering; UV, Ultraviolet light.
